# How to Stimulate Myocardial Regeneration in Adult Mammalian Heart: Existing Views and New Approaches

**DOI:** 10.1155/2020/7874109

**Published:** 2020-03-03

**Authors:** Galina Belostotskaya, Marc Hendrikx, Michael Galagudza, Sergey Suchkov

**Affiliations:** ^1^Sechenov Institute of Evolutionary Physiology and Biochemistry, The Group of Cytoanalysis, Russian Academy of Sciences, St. Petersburg, Russia; ^2^Almazov National Medical Research Centre, Institute of Experimental Medicine, St. Petersburg, Russia; ^3^Faculty of Medicine and Life Sciences, University of Hasselt, Belgium; ^4^ITMO University, St. Petersburg, Russia; ^5^EPMA, Brussels, Belgium; ^6^EU Center for Personalized Medicine, Sechenov University, Moscow, Russia; ^7^Department for Translational Medicine, Moscow Engineering Physical Institute (MEPhI), Moscow, Russia; ^8^PMC, Washington, DC, USA

## Abstract

Stem cell-based therapy has been considered as a promising option in the treatment of ischemic heart disease. Although stem cell administration resulted in the temporary improvement of myocardial contractility in the majority of studies, the formation of new cardiomyocytes within the injured myocardium has not been conclusively demonstrated. Consequently, the focus of research in the field has since shifted to stem cell-derived paracrine factors, including cytokines, growth factors, mRNA, and miRNA. Notably, both mRNA and miRNA can enter into the extracellular space either in soluble form or packed into membrane vesicles. Stem cell-derived paracrine factors have been shown to suppress inflammation and apoptosis, stimulate angiogenesis, and amplify the proliferation and differentiation of resident cardiac stem cells (CSCs). Such features have led to exosomes being considered as potential drug candidates affording myocardial regeneration. The search for chemical signals capable of stimulating cardiomyogenesis is ongoing despite continuous debates regarding the ability of mature cardiomyocytes to divide or dedifferentiate, transdifferentiation of other cells into cardiomyocytes, and the ability of CSCs to differentiate into cardiomyocytes. Future research is aimed at identifying novel cell candidates capable of differentiating into cardiomyocytes. The observation that CSCs can undergo intracellular development with the formation of “cell-in-cell structure” and subsequent release of transitory amplifying cells with the capacity to differentiate into cardiomyocytes may provide clues for stimulating regenerative cardiomyogenesis.

## 1. Introduction

The idea of extending the lifetime of the human heart has been fuelled by a series of major advances in transplantation and drug therapies. Nevertheless, myocardial infarction is characterized by the irreversible loss of cardiomyocytes because of their ischemic necrosis. Therefore, the need to re-establish the structural and functional features of native heart tissue represents a major challenge for the field of cardiac tissue engineering.

Over the past decade, stem cell (SC) transplantation has become a promising therapeutic strategy for treating acute and chronic myocardial ischemia. Both preclinical and clinical studies involving ES cells [1], induced pluripotent stem cells (iPSCs) [2], bone marrow cells (BMCs) [3, 4], mesenchymal stem cells (MSCs) [5], skeletal myoblasts (SMs) [6, 7], endothelial precursor cells (EPCs) [8], and cardiac stem cells (CSCs) [9, 10] showed significant albeit very moderate improvement in LV function following SC-based therapy. The ideal cell type for the treatment of heart disease should (i) improve heart function; (ii) create functional cardiac muscle and vasculature, integrated into the host tissue; (iii) be amenable to delivery by minimally invasive clinical methods; (iv) be available “off the shelf” as a standardized reagent; (v) be tolerated by the immune system; (vi) be safe oncologically; and (vii) circumvent societal ethical concerns.

Understanding how multipotent progenitors generate specific immature heart-cell lineages and form different heart-tissue components serves as a biotemplate for SC engineering to target cardiovascular regeneration. Accordingly, by utilising a primitive cell-fate map for the generation of such progenitors, the possibility also exists of regenerating the heart by transplanting specific progenitors derived from human SCs into patients with or at risk for cardiovascular disease. Towards this end, a clinically useful tissue-engineered graft needs to be designed to perform multiple different tasks including (i) reestablishment of the normal structure and function of injured myocardium across different size scales; (ii) functional integration with the host tissue; and (iii) remodelling in response to, e.g., environmental factors, growth, and aging. A “perfect” graft would balance these requirements to provide robust functionality on a long-term basis along with the capacity for vascularization.

In this review, our primary focus is on bioinspired strategies along with the knowledge gained to date regarding the challenges that remain to be addressed for engineered heart regeneration to become a clinical reality, within the framework of preventive and treatment strategies rooted in personalized and precision healthcare-based and translational resources. However, this field still lacks sufficiently conclusive results to support full-scale implementation of such approaches, as exemplified by the poor survival and long-term engraftment of transplanted cells. To address these issues, new strategies are being subjected to intense evaluation with regard to such applications, with the results suggesting that these strategies may improve the efficiency of next-generation SC-based therapies.

The lack of convincing clinical evidence to support the efficacy of CSC-based therapy of ischemic heart disease has questioned the initial hypothesis that new CMs are derived from CSCs and has led to the suggestion that myocardial renewal and regeneration in adult mammals are provided by dedifferentiation and subsequent division of mature CMs [11]. Although some researchers continue to discuss the contribution of resident CSCs to adult cardiomyogenesis [12, 13], the major part of scientific community has become increasingly skeptical regarding the involvement of CSCs in the generation of new CMs [14–17]. Moreover, the long-lasting debate on this issue, along with the lack of positive results, has resulted in the suspension of clinical trials on the use of c-kit^+^ CSCs and even denial of the very existence of c-kit^+^ CSCs in adult mammals [18].

In this review, we highlight the emerging approaches to afford regeneration of heart muscle *via* the phenomenon of intracellular development of CSCs accompanied by formation of cell-in-cell structures (CICSs) and the subsequent release of transitory amplifying cells (TACs) capable of differentiating into mature CMs. Such approaches open up new perspectives for promoting SC-induced cardiogenesis as a paradigm for regenerative medicine in the near future.

## 2. Cardiac Stem Cells (CSCs)

Comprehensive success has been accomplished in identifying several populations of resident CSCs (c-kit^+^, Sca-1^+^, Isl-1^+^, side-population (SP), cardiospheres, ALDH^+^ cells) in mammalian hearts and their availability in mature myocardium [10, 19]. Nevertheless, their successful use in myocardial regeneration procedures remains poor [2, 20]. Moreover, none of the SC types (including CSCs) satisfy the requirements needed for the treatment of cardiovascular diseases, such as improving heart function through myocardial cell regeneration without immune rejection or higher tumour formation risk [21]. Alternatively, the utilisation of several factors can improve the stability of transplanted CSCs in the unfavourable situation in the infarction zone. Coadministration of CSCs with MSCs [22] or the precultivation of c-kit^+^ CSCs, CD90^+^/CD105^+^ MSCs, and CD133^+^ endothelial progenitor cells, which led to adhesion and cluster formation [23], enhanced cell engraftment, the reduction of inflammation, and prevention of heart failure in animal experiments. Another novel approach was the fusion of CSCs and MSCs for hybrid cell generation, which decreased the infarction zone, unlike the simple mixture of these cell types [24].

In order to further improve the myocardial regeneration process, CSC preconditioning with growth factors, hypoxic shock, or proliferation and differentiation stimulants has also been applied. CSC genetic modification, which is used to improve specific features among SCs, represents another attempted advanced therapeutic method [25]. However, despite temporary signs of improvement with regard to cell survival, no significant heart regeneration was observed in any case.

### 2.1. Mechanism(s) of SC-Mediated Cardioreparative Effects

Numerous laboratory and clinical studies have shown that after performing cell therapy for congestive heart failure, improvement in left ventricular function occurred [26, 27]. In turn, absence of significant evidence of association of positive effects from SC usage and new CM formation in the damaged area allowed the positive impact of exogenous SCs injected into the damaged myocardium to be attributed to their influence on the myocardium as catalysed by humoral factors (*i.e.*, the paracrine effect) [28, 29]. This finding reduced interest towards cell therapy applications against heart diseases and turned the research direction towards the study of SC secretion factors as a potential alternative to SC therapy in mediating cardiac regeneration [30]. The host of factors synthetized within SCs and released into the extracellular milieu is usually referred to as the SC secretome. To date, MSC secretome analysis has identified >200 unique proteins [31]; notably, SC secretome components are not only confined to proteins but also include mRNA and miRNAs [32].

It has been shown that intravenous infusion of human MSCs reduces infarct size in mice via the anti-inflammatory protein TSG6; moreover, TSG6 alone was sufficient to provide a therapeutic effect [33]. This suggests that the therapeutic effects of MSCs may depend largely on their capacity to regulate inflammation and tissue homeostasis *via* an array of immunosuppressive factors, cytokines, growth factors, and differentiation factors [34]. These include IL-6, TGF-*β*, prostaglandin E2, HGF, EGF, FGF, PDGF, VEGF, IGF, stromal cell-derived factor 1, and, as discussed in more detail below, the tryptophan-catabolic enzyme IDO and NO, a product of iNOS. Cumulatively, these secreted factors may inhibit inflammatory responses, promote endothelial and fibroblast activities, and facilitate the proliferation and differentiation of progenitor cells in tissues *in situ* [35].

### 2.2. Role of the SC Secretome

SC secretome components can be secreted from the cell in different ways. Some utilise the classical secretory pathway involving a signal sequence that mediates individual protein secretion at the molecular level. Others are released through the process of exocytosis. Finally, many bioactive molecules are packed into membrane vesicles termed exosomes. At present, exosomes are considered as a potential alternative to stem cell therapy in mediating cardiac regeneration [30]. For example, mouse embryonic SC-derived exosomes exploit the regenerative power of ES cells without requiring their transplantation [36]. Exosomes of cardiosphere-derived cells (CDCs) inhibit the apoptosis and promote the proliferation of CMs, along with enhancing angiogenesis. Inhibition of exosome production blocks these regenerative processes. It has been shown that CDC exosomes contain a certain miRNA repertoire, with particular enrichment of miR-146a. Moreover, isolated administration of a miR-146a mimic reproduces some but not all of the benefits of CDC exosomes [37].

miRNA sequencing indicated that MSC-derived exosomes and MSCs themselves utilise similar mechanisms to enhance cardiac repair. In addition, it revealed that MSC-derived exosomes alone could be used to promote cardiac repair of the injured myocardium as a cardioprotective approach, along with the therapeutic potential of cardiac regeneration strategies [38–40]. The study of SC-derived exosomes has revealed that they contain growth factors, miRNA, and additional cytoprotective factors that aid in repairing and regenerating the damaged tissue. These exosomes exert antiapoptotic, antifibrotic, and proangiogenic function as well as enhancing cardiac differentiation, all of which are keys to repairing damaged tissue [41]. The provision of cell-free components, such as exosomes enriched in proteins, mRNAs, and miRNAs characteristic of parental SCs, therefore represents a potential approach for treating cardiovascular diseases [42]. Although the mechanisms by which exosomes improve cardiac function remain to be determined, these results support the concept that they constitute the main mediators of SC paracrine effects [32, 43] and that a paracrine mechanism is sufficient to elicit functional recovery in cell-based therapies for post-infarction-related chronic heart failure [44, 45].

To understand the biological role of extracellular vesicles (EVs), such as exosomes and microvesicles, and their involvement in myocardial regeneration, it is important to recognise that cardiac EV production increases in stress conditions, such as hypoxia, inflammation, or injury. In hypoxic conditions, cardiac EVs contain angiogenic and prosurvival factors. In acute myocardial infarction (AMI), damaged CMs produce EVs with increased content of angiogenic, anti-apoptotic, mitogenic, and growth factors [46]. These properties of exosomes support their likely utility as both biomarkers of cardiac damage and possible regulators of myocardium regeneration [46].

## 3. Innate Heart Regeneration

The concept of innate heart regeneration is based on the assumption that myocardium in principle is capable of rebuilding damaged cardiac muscle. However, this capability is highly variable and dependent on numerous as-yet-unidentified factors [47, 48]. Current research is focused on the identification of molecules that can stimulate cardiac regeneration through several mechanisms, including (i) induction of preexisting mature CM proliferation, (ii) cardiac fibroblasts being reprogrammed into CMs through direct transformation (*in vitro*), (iii) transdifferentiation of one of the various CM types, and (iv) activation of endogenous CSC differentiation into other myocardial cell types [49]. To date, the development of methods aiming towards the “rejuvenation” of dysfunctional myocardial cells appears quite promising in cardiology. In particular, miRNA is proposed as a regulator of myocardial cell activity. Moreover, some researchers have attempted to return mature CMs into a proliferation state [50] whereas others have provided evidence that miRNA can stimulate endogenous CSC differentiation [51]. Specifically, miR-590 and miR-199a were shown to induce DNA synthesis and cytokinesis in neonatal CMs in both mice and rats [52]. These miRNAs effected significant stimulation of heart regeneration and significantly improved cardiac function following myocardial infarction (MI) in mice. In comparison, miR-15 showed activity in mice only at one week after birth, which correlates with the rapid loss of myocardial regeneration ability. Notably, however, miR-15 inhibition improved cardiac function in mice after damage, thereby demonstrating its potential as a therapeutic agent [53].

The use of unique combinations of cardiospecific transcription factors, miRNAs, and/or chemical molecules is currently being considered for transdifferentiation of fibroblasts into CMs [49]. Moreover, it was established that transdifferentiation using Gata4, Mef2c, and Tbx5 is direct and does not yield multipotent cardiac precursors [54]. To activate resident CSCs, the use of metabolic programming has been suggested as this can change the metabolism of CSCs in accordance with external factors that originate from infarction or other cardiac pathologies [55].

Notably, studies of cardiomyogenesis-related stimuli are being performed concomitant with ongoing debates regarding the possibility of dedifferentiation and division of mature CMs and the ability of resident CSCs to differentiate into mature CMs in the adult damaged heart [47]. Although initially both mature CMs and resident CSCs had been considered equally important as a source of new CMs [56, 57], the critical analysis of data obtained by Porrello et al. [58] in neonatal mice together with the data of other authors in adult zebrafish [59, 60] has raised questions regarding this viewpoint. Further studies supported that new CMs are exclusively produced by means of adult CM dedifferentiation with subsequent proliferation [61–63].

Accordingly, recent studies explored the potential of different approaches capable of stimulating cardiomyogenesis from mature CMs. For example, Ma et al. [64] performed in-depth investigation of protein dynamics during heart regeneration of zebrafish using mass spectrometry. In comparison, Tahara et al. [65] studied the potential effects of paracrine signals of epicardial and endocardial cells on cardiomyocyte proliferation in zebrafish, whereas Foglia and Poss [61] along with Galdos et al. [66] attempted to approach the problem using heart development as a model. These authors compared two different biological principles of cardiomyogenesis: the lifelong ability to generate new CMs in zebrafish and CM formation limited to 5 days after birth in mammals. Nevertheless, despite the lack of a coherent scheme of myocardial regeneration, these studies shed some light on the issue. For example, it was established that a link exists between the lack of telomerase activity and concomitant telomere dysfunction, which serve as natural barriers to cardiomyocyte proliferation and cardiac regeneration [67]. In addition, p53 signalling was inhibited during heart regeneration [64]. Furthermore, it was shown that the regeneration of damaged myocardium is mediated only by CMs that are adjacent to the injury area [63] whereas inflammation [68] and ROS promote regeneration [69]. Therefore, these findings might together suggest that prolonged hypoxia is able to contribute to myocardial regeneration through the reprogramming of adult mammalian [70] and zebrafish [71] CMs.

In this regard, it is worth noting that hypoxia [72], ischemia [73], and infarction [74] all stimulate the appearance of small cardiac-positive cells in the mammalian myocardium, which can proliferate and differentiate leading to the formation of new CMs. When analysing these data, Kimura et al. [72] and Nakada et al. [75] suggested that inflammation and hypoxia promoted the division of mature CMs despite the fact that proliferating cells have an actual size of approximately 10–18 *μ*m, which is much smaller than that of mature CMs (*L* > 50 *μ*m). However, the issue of CM proliferation is also controversial. In particular, it was established that systemic hypoxemia could alleviate oxidative DNA damage, thereby inducing CM proliferation in adult mammals [75, 76]. Conversely, Tong et al. [77] previously showed that hypoxia causes a significant reduction in cardiomyocyte Ki-67 expression and BrdU incorporation in foetal rat hearts, demonstrating the inhibitory effect of hypoxia on CM proliferation in the developing heart.

### 3.1. Intracellular Development of CSCs

As the existing concepts fail to provide clear understanding of the observed phenomena, we suggested a new model based on the results obtained in our laboratory. By studying the behaviour of mammalian CSCs, we showed that the formation of new CMs from resident CSCs occurs through colony formation [78] and consequent to their intracellular development inside the CMs, forming cell-in-cell structures (CICSs) [79]. Both pathways of cardiomyogenesis were initially discovered in newborn rat myocardium cell culture and were then confirmed by analysing freshly isolated (*ex vivo*) myocardial cell suspensions from rats of varying ages, mature specific pathogen free (SPF) mice, a one-year-old bull, and a human [80]. In both cases, CSC proliferation terminated through the formation of TACs inside either colonies or CICSs, respectively. It was shown that intracellular development culminates in CICS breakage, which leads to the release of approximately 200 TACs. Free TACs with *L* = 10–12 *μ*m reserve their proliferative potential and differentiate into mature CMs upon exiting the CICSs. The imitation of ischemia (e.g., *via* hypoxia or acidosis) in *in vitro* experiments led to 5- to 10-fold increase in CICS amount and suppressed colony cell development [80].

### 3.2. The Role of Hypoxia in Cardiomyogenesis and Myocardial Regeneration

The comparison of our data with those obtained by other groups shows that hypoxia exerts dual effects on cardiomyogenesis. First, it prompts intracellular development of CSCs, which is evident from the increased number of CICSs. Upon maturation and rupture, CICSs release significant amounts of proliferating TACs [79, 80]; we presume that the same biological phenomenon was found by other groups, showing the appearance of small dividing cells after hypoxia, ischemia, and infarction [72–74]. In addition, the data of Nakada et al. [75] showed that 2-week hypoxemia (7% О_2_) imposed 1 week after MI has resulted in a burst of myocardial cell proliferation. These data point to an important conclusion that high myocardial regenerative capacity observed in adult zebrafishes and newts, as well as in mammalian embryos and newborn mammals, is explained by the intensive proliferation of their CMs in the hypoxic environment [71, 76].

It is known that hypoxia inhibits the proliferation of cardiac cells necessary to ensure embryonic and neonatal cardiomyogenesis [77]. Therefore, it might be speculated that hypoxia could be used to promote myocardial regeneration through the stimulation of intracellular development of CSCs concomitant with CICS formation, although it might be detrimental for the proliferation of TACs and their cardiac differentiation at the moment of massive TAC release from CICSs.

### 3.3. New Perspectives for Stimulating Regenerative Cardiomyogenesis

**Figure 1:**
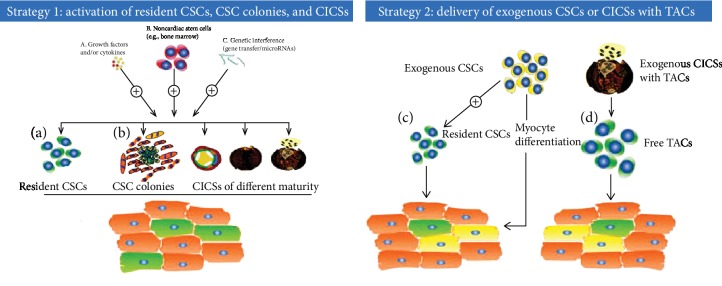
Novel strategies for CSC-based myocardial repair. Schematic overviews of current strategies used to make use of CSCs for myocardial repair are shown in a compilation of previously published figures [12, 13] with some modifications provided by us. Strategy 1 is based on activation of endogenous CSCs by various means, e.g., (a) growth factors, (b) noncardiac stem cells, or (c) gene therapy. Strategy 1(a): upon activation, resident endogenous CSCs can proliferate and mature into newly formed cardiac myocytes (green cardiac myocytes). Strategy 1(b) is based on activation of endogenous CSC colonies and CSC CICSs with formation of TACs. Strategy 2(c) is based on the delivery of autologous CSCs that have been isolated from small myocardial biopsies and scaled up outside the patient to sufficient numbers. Exogenous CSCs are also shown to be capable of activating the local endogenous CSC compartment. Strategy 2(d) is based on the delivery of autologous CICSs that have been isolated from small myocardial biopsies and scaled up outside the patient to sufficient numbers. In addition, exogenously delivered CSCs and CICSs are hypothesized to mature and differentiate into functional cardiac myocytes (yellow cardiac myocytes) that are electromechanically coupled with the preexisting cardiac myocytes (orange cardiac myocytes) [12]. Abbreviations: CSC: cardiac stem/progenitor cell; CICS: cell-in-cell structure; TACs: transitory amplifying cells.

Despite recent skepticism about the role of CSCs in myocardial self-renewal and regeneration in adult mammals [14–16, 18], our data demonstrate that these cells are strongly involved in cardiac regeneration. We consider that the phenomenon of CSC-derived CICSs with TAC formation might constitute a valuable scenario for potential utilisation in preselecting approaches to construct unique products exclusively focused on CICSs and TACs while targeting a sensitive subset of myocardial cells to secure a differentiation program to obtain a final pool of mature CMs. Moreover, TACs are highly sensitised to respond to the stimuli being preselected and targeted and exhibit markedly increased proliferative activity in postischemia and posthypoxia periods. Therefore, these cells would gain a unique ability to be induced towards cardiodifferentiation and are consequently the promising candidates to be applied to stimulate cardiomyogenesis in heart failure. The potential approaches to stimulation of myocardial regeneration are summarized in Figure 1, which is a compilation of previously published figures [12, 13] with some modifications provided by us.

## 4. Conclusion

By comparing our results with others, we concluded that all previously described small cells likely actually constitute TACs that “go through cardiac school (education)” inside mature CMs. It is important to highlight that intracellular CSC development is associated not only with partial cardiomyogenic differentiation of their progeny but also by the decreased level of their stemness. This fact hampers the identification of daughter cells for TACs and also ignores that the small size of TACs may lead to their erroneous interpretation as proliferating CMs. In addition, free CSCs are almost nondetectable in the adult mammalian myocardium, which strongly contributed to the negation of their role in adult cardiomyogenesis. However, the identification and in-depth exploration of the phenomenon of intracellular CSC development have encouraged us to claim that CSCs are not just innocent bystanders but rather working tools in an adult heart, which form TACs that afford myocardium renewal during their lifespan. The results from Malliaras et al. [74] confirmed that upon myocardial damage, small cells (11.5 ± 3.7 *μ*m), which were named by the authors as “endogenous cardioblasts,” are activated, and their amount increases more than 10-fold following infarction. Moreover, they showed that genetically labelled cardioblasts expressed heart transcription factors and sarcomere proteins, exhibited spontaneous contractions, and formed mature CMs (*in vivo*) following injection into the recipient heart.

Therefore, we consider that the existence of CSC-derived TACs has been conclusively demonstrated and should be considered in models of cardiomyogenesis. The schemes shown in Figure 1 illustrate two different approaches to stimulation of cardiomyogenesis. The first approach deals with the activation of endogenous CM progenitors (innate myocardial regeneration), while the second approach is based on myocardial delivery of exogenous CSCs or CICSs capable of producing new CMs. We feel that the second strategy might be more effective in terms of the time needed for the formation of new CMs, specifically with heart-targeted delivery of CICSs. According to our hypothesis, new CMs could be more readily produced from TACs, representing the offspring of the initial CSC, which underwent sequential proliferation and partial differentiation inside CICS. An important factor is the maturity of CICS. In Figure 1 (Strategy 1), CICSs are presented in the form of three images corresponding to different levels of maturity and readiness to opening (left to right), from immature to ruptured CICS capable of releasing of more than 200 TACs. From this viewpoint, the use of both immature CICSs and free CSCs will require more than 2 weeks for production of TAC batch. In addition, exogenously administered CSCs will not necessarily immediately start formation of CICSs and colonies. Therefore, innate myocardial regeneration strategy might prove to be more attractive for the clinical use. It might be anticipated that the use of appropriate factors stimulating cardiomyogenesis based on resident CSCs via colonies or CICS formation will result in a more rapid myocardial regeneration.

Our data indicate that cardiomyogenic stimuli should be focused on TACs, rather than on CSCs or mature CMs. It is also clear that the proliferative activity of TACs is enhanced following ischemia and hypoxia and that their cardiomyogenic differentiation ability renders them as top candidates for therapeutic cardiomyogenesis in the ailing heart.

Nevertheless, extensive work is still required to generate powerful off-the-shelf SC therapeutics as the data from human studies are contradictory, overall showing modest to no therapeutic SC effects [27, 81, 82]. To overcome this discrepancy, a deeper understanding of heart disease and endogenous reparative mechanisms along with their interactions with SCs is urgently needed [83]. Towards this end, there is growing evidence that patient characteristics, such as individual disease state, comedications, and personalized risk factors, critically influence the therapeutic outcome of SC applications. This finding clearly demands the implementation of personalized cardiac SC therapies in which the selection of SC source, modification, and application is tailored to these individual characteristics [84]. Thus, prospective research should focus on the development of specific responder scores and the identification of prognostic biomarkers to identify patient cohorts who would benefit most from distinct SC treatments [85, 86]. In addition, higher standardization of study design and the establishment of a global open-access database for the registration and publication of preclinical and clinical trials would markedly improve the comparability and availability of obtained data [86, 87]. Together with personalized cell-based therapy (e.g., responders versus nonresponders), SCs might thus be able to fulfil the expectations of novel curative options for patients with and at risk for cardiac disease.

The heart is an extremely complex organ, and the techniques influencing its regeneration depend on many variables of nontrivial character. Physician scientists and practitioners are working hand-in-hand with SC scientists, biodesigners, and bioengineers and are likely to play a key role in developing the new paradigms for heart regeneration that appear imminent. In this review and discussion, we aim to establish a “case study” for the field of regenerative medicine using cardiovascular regeneration as a model suitable for further utilisation. We expect that advanced modalities that integrate cellular, bioengineering, and information (IT) technologies *via* clinical studies and translational applications as new consolidated entities will enhance the efficacy of cardiac cell therapy and further contribute to cardiac regenerative medicine.
